# Institutes in the Lead: Identifying Environmental Factors in Breast Cancer

**DOI:** 10.1289/ehp.124-A199

**Published:** 2016-11-01

**Authors:** Nate Seltenrich

**Affiliations:** Nate Seltenrich covers science and the environment from Petaluma, CA. His work has appeared in *High Country News*, *Sierra*, *Yale Environment 360*, *Earth Island Journal*, and other regional and national publications.

In a way, it all started in Long Island, New York. The year was 1993. An apparent cluster of breast cancer cases had been discovered in Nassau and Suffolk counties, and some residents worried that pesticide applications on former farmland could be to blame.^1^ They demanded an investigation. The U.S. Congress soon agreed and asked the National Institute of Environmental Health Sciences (NIEHS) and the National Cancer Institute (NCI) to research the potential role of environmental exposures in these cases.^1^ In the decades since, these institutes have conducted and funded countless studies on potential environmental risk factors for breast cancer.^2^

Breast cancer, like many cancers, is a challenging disease to understand thoroughly because its causes include both genetic and environmental factors. Breast cancer also takes many years to develop, making it difficult for researchers to identify environmental factors “after the fact” that might have contributed to the initiation of the cancer.

Breast tissue receives hormonal signals from several endocrine organs (including the placenta, ovaries, pancreas, and thyroid) and responds to a wide range of hormones (including estrogen, progesterone, insulin, and thyroxine).^3^ Breast cancer researchers today are interested in exposures not only to chemicals that pose a cancer risk by altering DNA (in other words, classical carcinogens) but also to substances that may act on the body in other ways, such as endocrine disruptors.

“Endocrine disruptors are not expected to act as carcinogens per se—they don’t necessarily cause mutations or formation of DNA adducts,” explains Suzanne Fenton, leader of the NIEHS intramural Reproductive Endocrinology Group. “Rather, they cause a shift in how the body responds to normal hormones that the body produces all the time.”

Over time, studies examining how chemicals might contribute to breast cancer initiation have become better focused and more hypothesis-driven, says Deborah Winn, deputy director of the NCI’s Division of Cancer Control and Population Sciences. “The technology’s better, our questions are sharper, and the evidence is mounting,” she says. “I think we’re getting closer to [understanding environmental factors in a way] that’s clearer and that we can say something definitive about.”^4^ ^,^ ^5^

## Long Island Breast Cancer Study Project

At the time of the hypothesized Long Island cluster, breast cancer incidence nationwide averaged about 130 diagnoses per 100,000 people per year.^6^ In New York state from 1988 to 1992, rates were below average, about 122 cases per 100,000. But incidence in Nassau and Suffolk counties during this period was considerably higher: 139 and 133 cases per 100,000, respectively.^1^

The NIEHS and the NCI established a research study called the Long Island Breast Cancer Study Project (LIBCSP) to find out why. This effort included five distinct case–control sub-studies designed to address specific environmental exposures while controlling for other risk factors, such as age and reproductive history. Their goal was to evaluate whether increased risk of breast cancer was associated with high-priority exposures including organochlorine pesticides (such as DDT and its metabolite DDE); polychlorinated biphenyls (PCBs, once widely deployed in electrical equipment); polyaromatic hydrocarbons (PAHs, by-products of incomplete combustion); and electromagnetic fields (such as those generated by power lines and electric blankets).

Investigators ultimately reported two significant associations, both of which came from the project’s largest sub-study, the Breast Cancer and the Environment on Long Island Study. One showed that the odds of breast cancer were 50% higher in women with the highest levels of PAH-DNA adducts (which are biomarkers of exposure),^7^ and the other that the odds of breast cancer were almost 3 times higher in women living within 1 mile of hazardous waste sites containing organochlorine pesticides versus those who lived farther away.^8^

Ideally, the predominately negative results would help reduce anxiety among women concerned about their own risk from environmental factors, Winn noted in a 2005 commentary.^1^ “Findings of no association that are obtained through rigorous research are important,” she wrote. “The LIBCSP studies were able to fairly conclusively rule out several suspected environmental agents.”

But some members of the advocacy community on Long Island remained convinced that other exposures were responsible for increased cancer rates. Clearly, the story was far from over. And many would argue it continues today, not just in Long Island but across the country.

When those first five studies ended, the LIBCSP did not disappear; it simply entered its next phase of research. At the helm was principal investigator Marilie Gammon, a professor of epidemiology at the University of North Carolina at Chapel Hill. She had headed the Breast Cancer and the Environment on Long Island sub-study from its start in 1993. In 2001 she added a follow-up survivorship study—one of the first to examine the potential influence of environmental factors on breast cancer mortality—and included molecular epidemiological studies that involved examining tissue samples collected during the original research. Over the decades the effort has generated approximately 130 publications, Gammon says—a number that reflects the diversity of exposures and factors studied and the breadth of the findings reported. She continues to lead its investigations into PAHs and other exposures.

## Breast Cancer and the Environment Research Centers

Even before results from the Long Island studies were published, the NIEHS sought a greater understanding of the role of the environment in breast cancer causation. As early as 1994, the institute called for new *in vivo* and *in vitro* research to assess how the timing of certain exposures might make the breast more susceptible to damage.^9^

“Scientists were thinking about the idea that early-life exposures were most important and that it was too difficult to determine retrospectively environmental exposures in women who were getting breast cancer today,” says Julia Brody, executive director and senior scientist for the Silent Spring Institute, a Massachusetts-based research organization that has studied links between breast cancer and the environment since 1994.^10^

More than 20 years later, that idea remains central to the field. Researchers now realize that timing and length of exposure are critical variables in determining potential environmental influences on breast cancer development. Two particularly sensitive and widely studied exposure windows include the prenatal period and pubertal transition.

The breast develops in stages occurring before birth and during puberty,^11^ opening up high-risk windows of vulnerability. The time when mammary tissue is developing in the fetus is critical for chemical exposure, Fenton explains, while the onset of puberty involves major changes in the tissues that make them more susceptible to disruption. After puberty, once the breast is fully formed, susceptibility to chemical insult appears to be lower.

Later, in 2002, the NIEHS convened a landmark workshop on the subject together with the NCI, the nonprofit National Breast Cancer Coalition, and advocacy groups.^12^ According to participant Gwen Collman, now director of the NIEHS Division of Extramural Research and Training, one question in particular came to researchers’ attention: How do exposures during and just before puberty affect disease risk later in life?

The workshop’s conclusions soon led to the formation of the federally funded Breast Cancer and the Environment Research Centers (BCERC). Administered by the NIEHS and the NCI over a 7-year span,^13^ the initiative created population-based research centers at the Fox Chase Comprehensive Cancer Center in Philadelphia, the University of Cincinnati, and the University of California, San Francisco, Helen Diller Family Comprehensive Cancer Center.^14^ A fourth center at Michigan State University was established for basic biology projects and testing chemicals in animal models.^15^

More than 1,200 girls aged 6–8 years enrolled in the BCERC study. Researchers followed them at regular intervals to evaluate relationships among diet and obesity, exposures to a variety of endocrine-disrupting and carcinogenic chemicals, and pubertal development. The effort was unique in its combination of bench science, epidemiology, and public participation via community members and advocacy groups in one comprehensive program. Today this transdisciplinary approach is a hallmark of the NIEHS’s work in the field.

The BCERC study eventually provided a rich set of findings showing what may happen in the body as a result of various environmental stressors during the pubertal window.^14^ ^,^ ^16^ ^,^ ^17^ It established that girls are developing breasts earlier than previously thought, which increases the length of time they are exposed to estrogen. It documented important differences related to race/ethnicity and body mass index. And it provided, for the first time, a catalog of exposures commonly experienced by girls of this age range, tied to everything from cosmetics to the built environment.

What it didn’t do, however—and indeed, was not designed to do—was translate these results to cancer risk. That task falls to risk assessors based on evidence from research as it accrues over time. And more was on the way from another major research program at the NIEHS that was starting up just as BCERC was winding down: the Sister Study.

## The Sister Study

In 1999, at the time the Sister Study was proposed,^18^ much of the research on breast cancer was retrospective, based on recall or estimation of previous exposures rather than actual measurements. It is difficult to establish causal relationships between exposures and outcomes in such cases, says Sister Study principal investigator Dale Sandler, chief of the Epidemiology Branch at the NIEHS.

Prospective studies, or those that measure actual exposures during critical developmental windows and evaluate disease risk later in life, would be more definitive. However, existing prospective cohort studies at that time were focused mainly on diet, lifestyle, or hormone use rather than the chemical environment.

Furthermore, Sandler says, because effects of environmental chemicals might be difficult to detect due to low levels of exposure, a prospective study of chemical exposures and breast cancer in the general population would require a large number of women. Such a study would take a long time, potentially decades, before enough women developed breast cancer that researchers could identify contributing factors in a statistically meaningful way.

The solution? Improve statistical power by studying women who have an above-average genetic risk of developing breast cancer—women whose sisters had already been diagnosed.

“If you have a sister or a daughter or a mother with breast cancer, you have on average twice the chance of getting breast cancer as someone else in the population,” Sandler says. “By focusing on sisters, we increased the likelihood that we could pick up relationships between genes, exposures, and breast cancer risk.”

Sandler discovered another strength of the design during recruitment for this study: women whose families had been affected by breast cancer were highly motivated to participate. The study surpassed its recruitment goal, ultimately enrolling more than 50,000 women in the United States and Puerto Rico between the ages of 35 and 74. The women allowed researchers inside their homes to collect samples of blood, urine, nail clippings, and house dust.

“We also collected comprehensive histories of medication use, occupational factors, and residential factors in addition to the usual questions about reproductive factors and lifestyle that most studies include,” Sandler adds, “plus information on trauma, stress, use of personal care products, and more.”

Today the study continues tracking the health of these women. Each year participants report any changes to their health, and every 3 years they complete detailed questionnaires about their health, lifestyle, and chemical exposures. In addition, the research team has taken advantage of technological advances in genomics and other areas to expand the usefulness of the study data as a resource for other breast cancer researchers.

The study has generated more than 65 papers since 2009.^19^ Some of the results to date have provided preliminary evidence that environmental exposures do contribute to breast cancer risk and that timing of exposure matters, although more work is needed to clarify the relationships. Survivors continue to be followed as well, permitting Sandler’s team to study how environment and genes contribute not only to the incidence of breast cancer but also to survivorship.

The investigators are also looking for previously unsuspected environmental risk factors for breast cancer, Sandler says. Only now, after roughly 3,000 new cases of breast cancer have been diagnosed since the women were enrolled, are investigators able to conduct the sorts of analyses they imagined from the start. “We’re now on the cusp of having a very large comprehensive database with which to generate new information on environment and breast cancer,” Sandler says.

## The Two Sister Study

The Sister Study offered such a powerful design that early in its development it inspired a spin-off. In addition to the main prospective study, Sandler’s co-investigator Clarice Weinberg began setting up a companion retrospective family-based study called the Two Sister Study. Weinberg is the former chief of the Biostatistics and Computational Biology Branch at the NIEHS.

The Two Sister Study focused on women with young-onset breast cancer (those diagnosed before age 50) and their families.^20^ “You can actually determine relative risk associated with genotypes using cases and their parents, but the cancer has to be relatively young-onset because you need most of the [participants’] parents to be alive,” Weinberg explains. Using a methodology developed for the study of young-onset conditions such as birth defects, the Two Sister Study could provide new insights into gene–environment interactions and gene variants related to breast cancer risk.

In 2008 Weinberg received a grant from the Susan G. Komen Foundation to recruit and enroll more than 1,400 women recently diagnosed with young-onset breast cancer, plus their breast cancer–free sister who was already enrolled in the Sister Study and one or both parents. Participants provided detailed information about family and medical history, diet, and occupation, as well as samples of house dust and DNA.^21^ The work is designed to study potential genetic risk factors as well as environmental agents, such as metals.

The Two Sister Study has produced 7 papers to date, including most significantly a genome-wide association study that investigates maternal factors and other genetic mechanisms in young-onset breast cancer.^22^ The researchers have also reported that reduced risk of young-onset breast cancer is associated with the unsuccessful use of ovulation stimulation as a fertility treatment^23^ and with hormone replacement with estrogen alone.^24^ These findings suggest that a younger woman’s response to hormones may be related to her risk.^21^

Still, as with the Sister Study, the best is likely yet to come. According to Weinberg, it could take another 10 years or so to fully analyze the retrospective data.

## Animal Studies

Another way to find answers is through animal studies, which are often used as a counterpart to case–control and cohort studies or in some cases as a first line of evidence. This approach, too, has grown considerably more sophisticated over the years. Along with university-based research funded by the NIEHS, the institute’s National Toxicology Program (NTP) has focused research efforts on the role of environmental chemicals in mammary tumor risk under Fenton’s direction.^25^

The NTP program benefits from its ability to conduct lifetime rodent studies, which are often prohibitively expensive for university labs. Lifetime studies with mice and rats make it possible to expose animals to low doses of endocrine-disrupting chemicals at levels and on timelines that mimic human lifetime exposures, and evaluate mammary gland development and tumor incidence later in life. These rodent studies capture in 2 years what it could take 70 years to study in people.

“We’re generating data that other people can’t afford to,” Fenton says. “There’s a lot that we don’t know about the chemicals that affect the breast. Some of the missing information may be mechanistic in nature, but we still don’t know which chemicals change the susceptibility of the breast to tumor formation. We know some chemicals that are bad actors, but we don’t have a very long or well-thought-out list, mostly because of the time and funding needed for these long-term studies.”

One of the lab’s most important tools is a “whole-mount” preparation for assessing mammary gland development in mice and rats. This entails removing the intact mammary gland from the animal and viewing it in one piece. Whole mounts allow researchers to view the tissue in 3 dimensions, as opposed to sectioned into thin slices on dozens of separate slides. The whole-mount method offers an inexpensive way to identify preneoplastic lesions, precursors of tumor formation.^26^ In recent years Fenton’s group has begun teaching the whole-mount method to other toxicologists and documenting it in the scientific literature, with the aim of helping researchers across the globe more efficiently assess mammary gland development in rodent mammal systems.

Fenton and her colleagues use whole mounts to assess mammary gland development following early-life and pubertal exposures to endocrine disruptors such as herbicides and their metabolites, surfactants, phenolic compounds used in food packaging, and lipophilic flame retardants and pollutants. One recent study by the group, for example, shows that prenatal exposure to the common household chemical perfluorooctanoic acid (PFOA) causes significant and persistent mammary developmental delays in mice.^27^

## Breast Cancer and the Environment Research Program

Between 2010 and 2014 the NIEHS funded a second phase of BCERC^13^ ^,^ ^28^ to further investigate factors as diverse as prenatal environmental exposures,^29^ high-fat diets during puberty,^30^ bisphenol A exposures,^31^ and interactions among genes and endocrine disruptors.^32^ Now called the Breast Cancer and the Environment Research Program (BCERP), this phase maintained a predominate but not exclusive focus on pubertal exposures and development, Collman explains, coupled with a greater emphasis on animal studies designed to confirm and clarify what researchers were seeing in humans.

With the launch of a third phase in 2015, researchers further expanded the program’s focus to include earlier and later exposures. For example, a newly funded study out of City of Hope, a California-based research and treatment center, is investigating whether exposures to endocrine-disrupting chemicals during the menopausal transition may mimic the effects of hormone therapy and promote the development of breast cancer.^33^

The latest BCERP studies also reveal a growing interest in breast density as an intermediate marker of breast cancer risk.^34^ “We know that high breast density is a major risk factor for breast cancer, and there may be similar pathways by which exposures are influencing density,” explains former NIEHS program lead Caroline Dilworth.

Michigan State University professor of physiology Sandra Haslam has conducted animal research under BCERP since its origins as BCERC in 2003, although she first began studying hormones, human health, and breast cancer with NIH support as far back as 1980. Today she serves as co–principal investigator of a study investigating a new chemical of potential concern: oxybenzone, a common ingredient in sunscreen.^35^

“In reviewing the literature, there were indications that oxybenzone might have estrogenic activity,” she says. “There have been several studies in both mice and rats. By itself it doesn’t cause cancer, and [other researchers] have just looked at a lifetime exposure, but they never looked at intermediate points or specific effects on target tissues like the breast.”

Five other studies in this latest round of grants seek to answer a wide range of questions that together illustrate how far the field has come and the challenges that remain. Researchers at the University of Massachusetts are studying how exposures to xenoestrogens (chemical compounds that mimic estrogen) may erode the protective effect of pregnancy on breast cancer development.^36^ Another group will explore whether pubertal exposure to 3 different endocrine-active compounds—PFOA, butyl benzyl phthalate, and zeranol, a fungal xenoestrogen—alters breast composition and susceptibility to breast cancer.^37^ Still other projects will investigate the impacts of metal and metalloid exposures on breast density^38^ and transgenerational impacts of prenatal exposures to PAHs.^39^

Outside BCERP, the NIEHS funds many other researchers with similar interests, including Maggie Louie, a biochemistry professor at Dominican University of California. She’s now investigating how chronic low-level cadmium exposures act at the molecular level to promote breast cancer development and progression. Her current studies build on previous work showing that cadmium, present in cigarettes, food, water, and some cosmetics, can cause more aggressive breast cancers.^40^

## The Next Step

Although breast cancer mortality rates have declined dramatically in recent decades, incidence rates today hover around the 130-per-100,000 mark first reached in the late 1980s.^6^ Furthermore, breast cancer also affects about 1.25 out of 100,000 American men today, 25% more than 30 years ago.^6^ This makes prevention a critical piece of the puzzle that has not yet been filled in.^41^ ^,^ ^42^

In 2008 Congress passed the Breast Cancer and Environmental Research Act, which among other provisions mandated the establishment of the Interagency Breast Cancer and Environmental Research Coordinating Committee (IBCERCC). In 2013 this interagency group of government, academic, and advocacy representatives issued recommendations for the future of breast cancer research.^43^ Their suggested number-one priority was prevention, a departure from the status quo emphasis on diagnosis and cure.

The committee pointed out that only about 10–11% of federally funded breast cancer research focuses on environmental contributions to breast cancer. “Breast cancer prevention is underfunded at the federal level in both research and public health programs, and future investments must focus on this area,” they wrote.^43^ “Enhanced investments would facilitate sustained coordination across research and regulatory agencies with the objective of reducing or eliminating harmful environmental exposures and modifying social and lifestyle factors implicated in breast cancer.”

Granted, investments in diagnosis and treatment have paid significant dividends. Breast cancer has a far better prognosis today than it did 3 decades ago. In 1986, 1 in 4 women diagnosed with breast cancer died from it, but in 2013, the latest year for which government statistics are available, that number was closer to 1 in 6.^44^

While similar progress has not yet been made in reducing incidence rates, many researchers believe we can get there through a fuller understanding of the complex environmental and genetic factors influencing breast cancer development. Science-based preventive measures could one day mean fewer cases of breast cancer on the front end, just as research into treatment has lessened its impact post-diagnosis.

“We’re never going to be totally free of every chemical in the environment that could be detrimental,” Haslam says, “but we can make more intelligent choices about the ones that we have more information about. Hopefully we can address enough causative factors that we can reduce risk.”
